# Trekstock RENEW: evaluation of a 12-week exercise referral programme for young adult cancer survivors delivered by a cancer charity

**DOI:** 10.1007/s00520-020-05373-5

**Published:** 2020-03-27

**Authors:** G. Pugh, N. Below, A. Fisher, J. Reynolds, S. Epstone

**Affiliations:** 1 Centre for Sports & Exercise Medicine, William Harvey Research Institute, School of Medicine & Dentistry, Queen Mary University of London, London, UK; 2 Department of Behavioural Science & Health, Institute of Epidemiology & Health Care, University College London, London, UK; 3 Trekstock, London, UK

**Keywords:** Young adult cancer survivors, Exercise programme, Physical activity

## Abstract

**Purpose:**

To evaluate the uptake and effect of RENEW, a 12-week exercise referral programme for young adult cancer survivors delivered by Trekstock, a UK-based cancer charity.

**Methods:**

The RENEW programme provides one-to-one individually tailored support from a level-4 cancer-rehabilitation-qualified gym instructor, free gym membership and access to information resources online. Objective and self-report data on cardiorespiratory function, strength, body composition, fatigue, sleep quality and general health-related quality of life (HRQoL) was collected from participants before the programme (week 0), immediately after (week 12) and 1 month later (week 16).

**Results:**

Forty-eight young adults (83% female; mean age, 29 years) with a history of cancer took part within the 12-week programme and completed the evaluation measures. Physical activity (PA) levels significantly increased following the programme and remained raised at follow-up. Improvements in physical function were significant: peak expiratory flow (mean change, 30.96, *p* = 0.003), sit-and-reach test (mean change, 6.55 ± 4.54, *p* < 0.0001), and 6-mine-walk test (mean change, 0.12 ± 0.04, *p* < 0.0001). No significant changes in BMI, weight or muscle mass were observed. Improvements in fatigue, sleep and HRQoL were observed across the programme and at follow-up (mean change, weeks 0–16; 8.04 ± 1.49 *p* < 0.01; 1.05 ± 0.49 *p* < 0.05; and − 0.9 ± 0.46 *p* = 0.051, respectively). Changes in self-efficacy to exercise and motivations to exercise were not observed at 12 weeks or at follow-up.

**Conclusions:**

Results suggest that the RENEW exercise referral programme has a positive impact upon some domains of physical function and well-being among young adult cancer survivors.

**Implication for cancer survivors:**

Exercise referral programmes delivered by charity organisations are one means by which PA behaviour change support may be widely disseminated to young adult cancer survivors. Health professionals and charitable bodies specialising in the care of young adults with cancer should look to address factors which prevent engagement and uptake of ‘real-world’ PA interventions such as the RENEW programme.

## Background

Systematic reviews and meta-analyses of high-quality randomised controlled trials demonstrate physical activity (PA) has a positive effect on a number of clinical, physical and psychosocial outcomes among cancer survivors [1–3]. Given the benefits of exercise for individuals with cancer, the World Cancer Research Fund (WCRF), American Cancer Society and American College of Sports Medicine (ACSM) recommend that cancer survivors reduce their sedentary time and aim to accumulate 150 min/week of moderate-intensity aerobic exercise (or 75 min/week of vigorous intensity aerobic exercise) and two strength training sessions per week. However, at present, the vast majority of studies have been conducted in controlled clinical environments (hospitals/academic institutions) among cancer survivors over the age of 50 diagnosed with breast, prostate or colorectal cancer [4, 5]. There is increasing recognition that PA plays a role in reducing cancer recurrence risk and mortality among young adult cancer survivors [4, 6]. and that efforts need to be made to translate PA interventions into real-world practice [7].

The term ‘young adult cancer survivor’ typically refers to an individual who is 18–39 years of age who has had a diagnosis of cancer [8]. This age range is distinct from a physiological and psychosocial perspective: young adults have passed puberty but have not yet experienced the effects of hormonal decline, immune response deterioration or organ dysfunction associated with older age and chronic health conditions [9]. Given that young adult cancer survivors will experience many decades more of survivorship than older adults, it has been widely acknowledged that sustainable and practical evidence-based survivorship programmes for young adult cancer survivors are required [10, 11].

Trekstock is a London-based charity which provides information and support programmes to help cancer survivors in their 20s and 30s deal with the emotional and physical impact of cancer. Based upon the evidence that physical activity is a means to enhance physical and psychosocial outcomes among individuals with cancer, Trekstock programmes predominantly centre around physical activity promotion. To address young adult cancer survivors’ need and desire for structured physical activity support [12, 13], Trekstock developed a 12-week exercise programme (RENEW) consisting of personal training support from a qualified level-4 cancer-rehabilitation instructor within a community-based setting, free gym membership and support materials available online via the Trekstock website. The delivery of an individually tailored yet structured physical activity support programme by a charitable organisation in a community-based gym setting is novel and addresses young adult cancer survivors need for PA interventions which foster independence and autonomy away from primary treatment centres [13].

The purpose of this evaluation is to investigate the effect of the RENEW exercise programme upon the health and well-being of young adult cancer survivors. The primary objective was to assess rates of uptake of and adherence to the programme. The secondary objective was to determine the effect of the programme on the (i) physical health and function, (ii) quality of life, sleep and fatigue, (iii) PA behaviour; and (iv) self-efficacy for, and motivation to, exercise.

## Methods

### Participants and recruitment

Participants were service users of Trekstock defined as young adults between the age of 18 and 39 years who have had a cancer diagnosis at any point within their lifetime. Between August 2018 and May 2019, advertisements for the RENEW programme were shared through the Trekstock network and in London-based cancer centres specialising in the treatment and care of young adults. If interested in taking part in the RENEW programme, participants were asked to complete a self-referral form via the Trekstock website. Trekstock then linked the participant to a level-4 cancer-rehabilitation personal trainer to commence the programme at a time convenient to themselves. To ensure safety of delivery, if a participant was in active cancer treatment, clearance from their medical team was required before they were linked with a personal trainer.

### RENEW exercise programme

Trekstock linked each participant to a level-4 cancer-rehabilitation personal trainer at the Central YMCA gym in London. Participants met with the personal trainer at 4 time points across the 12-week programme. At baseline (week 0, initial meeting), trainers worked with each participant to develop a tailored programme based on their age, cancer type and any comorbidities. Hour-long personal training sessions were held at time points selected by the participants and trainers. All sessions began with a warm-up, followed by aerobic and strength training exercise before a cool-down. The total cost incurred by Trekstock to deliver the programme in partnership with YMCA was £200 per person.

### Evaluation design

A quasi-experimental pre- and post-test design was adopted to evaluate the programme. It was not compulsory for young adults with cancer who were taking part in the RENEW programme to take part in the evaluation. Self-report data on physical activity behaviour, health related quality of life, sleep, fatigue, motivation and self-efficacy to exercise were collected at 3 time points pre-programme (baseline, week 0), immediately following delivery of the exercise programme (T1, week 12) and 1 month later (T2, week 16). Objective measures of physical health were taken by the personal trainer at baseline (week 0) and post-programme (T1, week 12). There was no incentive or compensation provided to participants for taking part in the programme or for completing the evaluation measures. Ethical approval for the evaluation was granted by the Queen Mary University Research Ethics Committee (reference: QMREC2018/48/006).

## Measures

### Demographic and health characteristics

Self-report data on age, ethnicity and educational/employment status was collected alongside data on current health status and cancer history (diagnosis, age at diagnosis, treatment received and current treatment status). Weight, height, resting blood pressure, resting heart rate, body fat mass and muscle mass data were collected by personal trainers who deliver the RENEW programme at baseline and post-programme. Body fat mass and muscle mass were assessed using bioelectrical impedance analysis (YMCA, body composition scanner).

### Functional health

Data on peak expiratory flow (PEF), sit-and-reach performance and 6-min walk test (6MWT) performance were collected by personal trainers who deliver the RENEW programme. These are valid, reliable field based measures of lung function, flexibility and cardiorespiratory fitness [14, 15]. The 6MWT is considered a valid measure of cardiorespiratory fitness, correlation coefficient = 0.67 between 6MWT and oxygen consumption (VO_2_) peak [16].

### Physical activity and sedentary behaviour

The Godin Leisure Time Exercise Questionnaire (GLTEQ) was used to assess physical activity. Participants were asked to report the frequency and duration of mild, moderate and strenuous exercise they engage in over typical week. The GLTEQ has been used in similar studies of TYA cancer survivors [17, 18] and has previously demonstrated reliability and validity within the oncology research setting [19]. A single item from the International Physical Activity Questionnaire (IPAQ) regarding the amount of time spent sitting down in the last week and two items taken from the National Health and Nutrition Examination Survey (NHANES) [20] were used to assess computer and television viewing as proxy measures of sedentary behaviour.

### Self-efficacy and self-determined motivation to exercise

Physical activity self-efficacy was measured using the Self-Efficacy for Exercise (SEE) scale [21] a revision of McAuley’s (1990) self-efficacy barriers to exercise measure, which is made up of 13 items relating to the ability to continue exercising in the face of barriers to exercise. These were rated on an 11-point scale (0 = not confident, 10 = very confident). The information gathered by this standardised subjective measure is descriptive of participants’ level of confidence to be active with a higher score indicating greater self-efficacy. The Behavioural Regulation in Exercise Questionnaire (BREQ) was used to assess participants’ motivation to exercise [22]. The BREQ2 measures external, introjected, identified and intrinsic forms of regulation of exercise behaviour based on Deci and Ryan’s (1985, 1991) hierarchical model of extrinsic and intrinsic motivation. Each domain of the BREQ was scored on a scale of 1–6 with a high score indicating more dominance of that domain of motivation. Physical activity self-efficacy was measured using the Self-Efficacy for Exercise (SEE) scale [21] which is made up of thirteen items relating to the ability to continue exercising in the face of barriers to exercise. These were rated on an 11-point scale (0 = not confident, 10 = very confident). The information gathered by this standardised subjective measure is descriptive of participants’ level of confidence to be active with a higher score indicating greater self-efficacy. The Behavioural Regulation In Exercise Questionnaire (BREQ) was used to assess participants’ motivation to exercise [22]. The BREQ2 measures external, introjected, identified and intrinsic forms of regulation of exercise behaviour. Each domain of the BREQ was scored on a scale of 1–6 with a high score indicating more dominance of that domain of motivation.

### Health-related quality of life

Health-related quality of life (HRQoL) was measured using the five-item EuroQoL-5, a valid (Cronbachs alpha = 0.71) and reliable (*r* > 0.7) measure of health status commonly used in cancer research [23]. The five-item questionnaire assesses mobility, self-care, daily activities, pain and anxiety/depression on a 5-point Likert scale. Each item is scored to provide a global score ranging from 5 to 25; higher scores indicate poorer HQQoL. The EuroQoL-5 has previously been used among TYA cancer survivors [24].

### Fatigue

Fatigue was measured using the Functional Assessment of Chronic Illness Therapy Fatigue (FACIT-F) questionnaire which assess fatigue and its’ impact on physical and psychosocial well-being using 13 items on a 5-point Likert scale. The FACIT-F is widely used among cancer patients [25] and has previously been used in studies of TYA cancer survivors [24]. Scoring of the FACIT-F measure obtains a global score ranging from zero to 52, with a higher score indicating better quality of life [26].

### Sleep quality

The Pittsburgh Sleep Quality Index (PSQI) was used to assess seven dimensions of global sleep quality over the previous month. The PSQI is a 19-item self-report measure which identifies subjective sleep quality, sleep latency, sleep duration, habitual sleep efficiency, sleep disturbances, sleep medication and daytime function. The PSQI has been validated for use among cancer patients (Cronbach’s α = 0.81) and shows good reliability (*r* = 0.85) [27]. A combined global sleep score was calculated for each participant ranging from 0 to 21, with greater scores indicating poorer sleep quality.

### Statistical analyses

Descriptive statistics (M, SD, *n*, %) were calculated in order to summarise this data on participant characteristics, programme retention and attrition. Continuous data are presented as means and standard deviation; nominal and ordinal data are presented as frequencies and percentages. All data were checked for distribution and evaluated for normality using the Shapiro-Wilk test. Paired sample *t* tests were used to examine changes in physical function and health variables from baseline to post-programme. For non-normally distributed variables, non-parametric Wilcoxon Signed Rank tests were used. To examine differences over time repeated measures analyses of variance (rANOVAs) were calculated with factors time (baseline vs. T1 vs. T2). Prior to these analyses, missing data values were imputed using the Markov-Chain Monte-Carlo (MCMC) multiple imputation method. All statistical analysis was conducted using SPSS software (version 25.0) with statistical significance set at *p* < 0.05.

## Results

### Rate of uptake and adherence to the programme

**Fig. 1:**
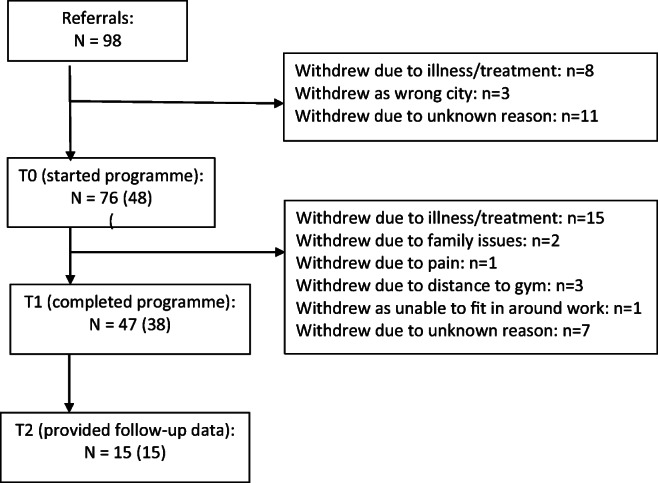
Flow of participants. Data provided in brackets refers to those who completed the evaluation outcome measures

As displayed in Fig. 1, a total of 98 participant referrals to the RENEW programme were made. Of these, 76 proceeded to commence the programme of whom 48 opted to take part in the evaluation. Ten participants who took part in the evaluation did not complete the 12-week programme for reasons listed within the figure. Of the 38 participants who provided data at T1, 15 provided data at T2. No adverse events were recorded during the RENEW programme among either those who chose to take part in the evaluation (*n* = 48) or those who took part in the programme in general (*n* = 76).

### Participant characteristics

Table 1 summarises participant demographic and health characteristics. The majority of participants (*n* = 48, mean age, 29 years) were female (83%, *n* = 40), white (81%, *n* = 39) and educated to a minimum of a bachelor’s degree (58%, *n* = 28). The most common cancer diagnosis was lymphoma (18%, *n* = 9) followed by breast cancer (16%, *n* = 8) and leukaemia (12%, *n* = 6). Most had completed their main cancer treatment (66%, *n* = 32) but were within the first 12 months of survivorship or undergoing active surveillance (37%, *n* = 18). At baseline, 2 participants were classified as underweight (BMI < 18.5), eight as overweight (BMI 25–30) and seven as obese (BMI > 30).

**Table 1:** Participant characteristics and health demographics

	Number	Mean (SD) or %
General demographics		
Age (years)	48	29.04 (5.37)
BMI (kg/m^2^)	48	26.09 (6.86)
Range (kg/m^2^)		16.8–45.7
Gender		
Female	40	83.3%
Male	8	16.7%
Ethnicity		
White	39	81.3%
Mixed	2	4.2%
Bangladeshi	1	2.1%
Indian	3	6.3%
Black African	2	4.2%
Black Caribbean	1	2.1%
Education		
GCSE/school certificate	2	4.2%
Vocational qualifications	1	2.1%
A-Level/high school certificate oe	6	12.5%
Bachelor degree oe	18	37.5%
Masters/PhD/PGCE oe	10	20.8%
Employment		
Employed full time	10	20.8%
Employed part time	4	8.3%
Self-employed	7	14.6%
Full-time education	4	8.3%
Part-time education	1	2.1%
Unemployed, looking for work	2	4.2%
Voluntary work	1	2.1%
Unable/too ill to work	8	16.7%
Marital status		
Married/living with partner	12	25%
In a relationship	5	10.4%
Single	21	43.8%
Divorced	1	2.1%
Prefer not to say	1	2.1%
Living arrangements		
Living with partner	10	20.8%
Living with family	16	33.3%
Living with friends	5	22.7%
Living alone	6	12.5%
Cancer type		
Breast	8	16.7%
Lymphoma	9	18.8%
Leukaemia	6	12.5%
Bowel	4	8.3%
Bone	2	4.2%
Carcinoma	2	4.2%
Brain	1	2.1%
Ovarian	1	2.1%
Womb	2	4.2%
Soft tissue sarcoma	1	2.1%
Lung	1	2.1%
Cervical	1	2.1%
Testicular	2	4.2%
Other	8	16.6%
Cancer stage		
0	1	2.1%
1	7	14.6%
2	12	25%
3	13	16.7%
4	5	10.4%
Do not know	12	25%
Treatment status		
Did not answer	4	8.4%
Completed	32	66.7%
Active	12	25%
Time since treatment		
On active treatment	7	26.9%
1–3 months	1	3.8%
4–11 months	10	38.5%
1–5 years	8	30.8%
Treatment received		
Chemotherapy	37	77.1%
Radiotherapy	21	43.8%
Surgery	28	58.3%
Hormone therapy	9	18.8%
Other	11	22.9%

Where percentage < 100 denotes missing data; where participants with > 100% had the option to select more than one answer

### Effect on physical function and health

Table 2 displays pre- and post-changes in physical function following the RENEW programme. Significant improvements in lung function (mean PEF change, 49.03 ± 8.44; *p* < 0.000) fitness (6MWT distance, 0.13 ± 0.03; *p* = 0.00) and flexibility (mean S&R change, 6.67 ± 0.95, *p* = 0.00) were observed. Overall no significant change was observed in the weight status, fat mass, muscle mass, resting blood pressure or resting HR of participants.

**Table 2:** Change in physical activity behaviour (mean ± SE)

Outcome	Baseline	Post-programme	Follow-up	T0–T1	*p* value	T1–T2	*p* value	T0–T2	*p* value
Physical activity (GLTEQ)	37.94 ± 3.07	56.35 ± 1.88	60.00 ± 1.56	18.40 ± 3.25	0.000	3.64 ± 2.19	0.099	22.05 ± 3.34	0.000
Frequency of PA per week									
Mild	4.40 ± 2.47	6.25 ± 2.93	6.00 ± 2.44	− 2.13 ± 2.73	0.004	0.11 ± 1.05	0.760	2.38 ± 2.84	0.011
Moderate	3.67 ± 2.26	4.15 ± 2.91	4.42 ± 1.41	− 0.52 ± 3.25	0.489	0.44 ± 1.42	0.377	0.05 ± 2.2	0.409
Vigorous	1.03 ± 1.54	2.00 ± 1.16	2.26 ± 1.23	− 0.71 ± 1.34	0.024	0.11 ± 0.60	0.594	1.03 ± 1.39	0.020
Sedentary behaviour^a^	13.3 ± 4.6	15.0 ± 10.9	16.2 ± 12.0	0.06 ± 8.16	0.953	1.23 ± 2.49	0.249	0.65 ± 5.09	0.574

^a^Total hours of sitting time, TV viewing and computer use per day

### Change in physical activity

Change in physical activity levels are show in Table 3. At baseline, 79% (*n* = 38) of participants were classed as active (GLTEQ Score > 24). There was a significant increase in GLTEQ score from baseline to post-programme (mean change T0–T1, 18.40 ± 3.25; mean change T0–T2, 22.05 ± 3.34; *p* < 0.01). Frequency of mild, moderate and vigorous intensity exercise increased across the programme and remained stable at follow-up.

**Table 3:** Change in physical function (mean ± SE)

Outcome	Baseline	Post-programme	Mean diff ± SD	*p* value
			T0–T1	
Resting systolic BP	113.66 ± 1.57	113.77 ± 1.54	0.10 ± 1.42	0.944
Resting diastolic BP	76.93 ± 1.28	79.23 ± 1.15	2.30 ± 1.06	0.031
Resting HR	80.23 ± 1.36	79.36 ± 1.25	0.65 ± 1.07	0.543
Peak expiratory flow	407.66 ± 10.42	456.70 ± 12.45	49.03 ± 8.44	0.000
Sit and reach (cm)	13.1 ± 1.80	19.8 ± 1.64	6.76 ± 0.95	0.000
6 min walk test (km)	0.525 ± 0.29	0.65 ± 0.28	0.13 ± 0.03	0.000
Muscle mass	26.33 ± 0.05	26.85 ± 0.63	0.51 ± 0.38	0.177
Fat mass	25.42 ± 1.88	25.34 ± 1.85	0.07 ± 0.65	0.909
Weight (kg)	73.48 ± 2.43	74.01 ± 2.39	− 0.52 ± 0.66	0.427
BMI	26.09 ± 0.88	26.17 ± 0.87	− 0.08 ± 0.38	0.834

### Effect on self-efficacy and motivation to exercise

Changes in self-efficacy and motivation are displayed in Table 4. Overall there was no significant change in self-efficacy or self-determined exercise motivation from baseline to follow-up (*p* < 0.05). A small decrease in external regulation was observed and small increase in intrinsic regulation was observed across the 12-week programme.

### Change in sleep, fatigue and health-related quality of life

Changes in sleep, fatigue and HRQoL are shown in Table 5. There was a significant improvement in participant fatigue scores across the programme from baseline to follow up (mean change T0–T2: 8.04 ± 1.49; *p* < 0.05). Fatigue scores significantly improved throughout the 12 week intervention period (T0–T1, 10.1 ± 1.45; *p* < 0.005) and plateaued at follow-up (T1–T2, 2.1 ± 1.27; *p* = 0.100) HRQoL and sleep quality significantly improved throughout the programme (mean change T0–T2, 0.9 ± 0.46; *p* < 0.05 and 1.05 ± 0.49; *p* = 0.051 respectively).

**Table 4:** Self-efficacy and motivation (mean ± SD)

Outcome	Baseline	Post-programme	Follow-up	T0–T1	*p* value	T1–T2	*p* value	T2–T0	*p* value
Self-efficacy^a^	56.82 ± 18.54	54.4 ± 21.03±	57.30 ± 24.50	4.06 ± 3.5	0.247	0.11 ± 2.51	0.965	4.17 ± 2.75	0.129
Motivation and behavioural regulation^b^									
Amotivation: lacking any intention to engage in PA.	3.78 ± 0.72	3.53 ± 0.12	3.26 ± 0.15	0.25 ± 0.19	0.075	0.26 ± 0.19	0.169	0.52 ± 0.17	0.004
External regulation: engaging in PA only in order to satisfy external pressures or to achieve externally imposed rewards.	3.09 ± 0.13	3.01 ± 0.143	2.97 ± 0.17	0.07 ± 0.18	0.684	0.04 ± 0.23	0.842	0.12 ± 0.24	0.618
Introjected regulation: internalisation of external controls, which are then applied through self-imposed pressures in order to avoid guilt or maintain self-esteem.	1.72 ± 0.15	1.95 ± 0.20	1.53 ± 0.11	0.23 ± 0.21	0.151	0.18 ± 0.21	0.398	0.42 ± 0.27	0.271
Identified regulation: conscious acceptance of PA as being important in order to achieve personally valued outcomes.	1.02 ± 0.11	1.19 ± 0.17	1.01 ± 0.09	0.17 ± 0.20	0.406	0.17 ± 0.50	0.208	0.00 ± 0.14	0.956
Intrinsic regulation: taking part in PA for the enjoyment and satisfaction inherent in engaging in the behaviour itself.	1.53 ± 0.16	1.44 ± 0.17	1.64 ± 0.15	0.09 ± 0.21	0.439	0.19 ± 0.23	0.831	0.09 ± 0.19	0.497

^a^SEE score range is from 0 to 90 (0 indicating no confidence to exercise for each outcome; 90 indicating high confidence to exercise when faced with each listed barrier)

^b^The BREQ is scored on a scale of 1–6. For each domain, a higher score indicates more dominance

**Table 5:** Change in sleep, fatigue and HRQoL

	Baseline	Post-programme	Follow-up	T0–T1	*p* value	T1–T2	*p* value	T0–T2	*p* value
	Mean ± SE	Mean ± SE	Mean ± SE						
Fatigue (FACIT-F)	23.17 ± 1.19	33.34 ± 1.11	31.22 ± 0.96	10.1 ± 1.45	< 0.005	21. ±1.27	0.100	8.04 ± 1.49	< 0.01
Sleep (PSQI)	7.15 ± 0.36	8.35 ± 0.38	6.10 ± 0.35	1.2 ± 0.47	0.013	2.25 ± 0.56	0.000	1.05 ± 0.49	0.034
HRQoL (EURO-5QD)	9.07 ± 0.44	8.48 ± 0.38	8.16 ± 0.24	0.59 ± 0.45	0.191	− 0.31 ± 0.46	0.503	− 0.9 ± 0.46	0.051

*T0*, baseline; *T1*, 12 weeks; *T2*, 16 weeks

## Discussion

There is increasing evidence that remaining active during and after treatment has a number of physical and psychosocial benefits for cancer patients and survivors. However, there are very few tailored interventions available to support young adult cancer survivors aged between 18 and 39 years to either increase or maintain their activity levels following a cancer diagnosis. The RENEW programme delivered by Trekstock was found to be effective in improving the PA levels, fitness, physical function, HRQoL, sleep quality and fatigue levels of young adult diagnosed with cancer. Benefits were observed among both cancer patient (those receiving treatment) and survivors (those who had completed treatment).

Interest in programme participation was high with 98 young adults expressing interest in the 9-month recruitment window; 77% of young adults who signed up for the RENEW programme attended at least one exercise session with 61% of these young adults completing the full 12-week programme. This rate of uptake is notably higher than many exercise intervention studies conducted in the hospital setting or academic setting among similar age groups [28, 29]. It is likely that the delivery of the programme by a trusted charity organisation, the ease of the referral process and the guarantee of personally tailored exercise support alongside free gym membership underpinned young adults’ interest in enrolling. However, 22% of young adults who came forward as being interested in the RENEW exercise programme did not initiate the programme; the primary reason for drop out either prior to the programme or during the 12-week RENEW period was illness. Whilst no negative adverse events were recorded during the RENEW programme and the ACSM exercise guidelines state medical assessment for cancer survivors before initiating an exercise programme is unnecessary due to the high benefit/low risk [30], the finding that illness was the primary cause of drop-out reiterates the importance of qualified exercise professionals adapting programmes to suit the health status of each individual cancer patient/survivor. Akin to existing intervention studies within the literature, the majority of individuals who enrolled to the RENEW programme were female, white British and highly educated. This suggests that effort need to be made to ensure that advertisements and access to the RENEW programme reach all young adults with cancer who require support. User engagement studies are currently being carried out to investigate young adult male cancer survivors’ viewpoint of Trekstock services.

The RENEW programme demonstrated a positive impact on the physical activity levels of young adult cancer survivors. Total amount of physical activity and frequency of mild, moderate and vigorous exercise generally increased across the programme duration. This is encouraging and supports the UK National Institute for Health and Care Excellence position that structured exercise referral schemes delivered and developed by qualified professionals (such as L4 Cancer Rehab trainers) can address inactivity among people with chronic disease [31]. Thought towards different models of exercise programme delivery for young adults with cancer, especially those living in rural areas without easy access to gym facilities, is required. Whilst digital interventions conducted among adolescent and young adult cancer survivors have demonstrated promising outcomes, there is currently little evidence of the economic value of implementing digital interventions on a wide-scale in AYA cancer services [32].

Significant improvements were observed in some domains of physical function (lung function, fitness and flexibility). This is promising and reflective of existing data from intervention studies conducted among adolescent and childhood cancer survivors of a similar age [28, 29, 33, 34]. The improvements in chronic fatigue scores, sleep quality and HRQoL are also encouraging and reiterate the benefit of physical activity upon the psychosocial health and well-being of young adults with cancer [35, 36].

Among adult cancer survivors, high self-efficacy to exercise has been found to predict better quality of life, lower levels of cancer-related fatigue and higher exercise intervention adherence [37, 38]. Data from the Wellspring Cancer Exercise Programme in Ontario, Canada demonstrate that self-efficacy to exercise can affect exercise programme outcomes with those who have higher levels of self-efficacy demonstrating greater improvements in HRQoL and fatigue [38]. Participants within this study demonstrated high levels of exercise self-efficacy and motivation to exercise at baseline indicating that the RENEW programme failed to reach those who lack the confidence to engage in or commit to an exercise programme. In accordance with existing research [38], advertising and highlighting the features of the RENEW programme which are known to improve self-efficacy to exercise (e.g. personalised exercise plan, autonomy to choose exercise modality and positive feedback upon progress) may nudge some young adults to engage in the programme. Providing young adults cancer survivors with the opportunity to see other cancer survivors of a similar age who have similar physical limitations complete exercise successfully is a further solution by which young adults with low self-efficacy and motivation to exercise may be better engaged. Data from the evaluation of the Trekstock Meet and Move programme confirm this notion and indicate that 1-day events which provide an opportunity for young adults with cancer to meet others are effective at improving confidence to be active [39].

### Strengths and limitations

A major strength of this work is that it is one of very few evaluations of community-based exercise programmes delivered by a charitable organisation which uses objective outcome measures. Despite the strengths of the RENEW evaluation (e.g. prospective repeated measures design, independent data collection and participant retention) several limitations must be noted when interpreting the results of the data presented. First, the generalisability of the results is limited due to the prevalence of highly educated white females within the sample. Secondly, there is a high likelihood that response bias in that young adults who participated in the programme are likely to report different outcomes (specifically for self-efficacy and motivation) than those who elected not to enrol. Although the sample within the study is comparable with other physical activity intervention studies conducted among young adult cancer survivors (e.g. [40]), it is likely that there was inadequate power to detect a significant difference between time-points for some outcome measures.

### Implications and future directions

Advancements in the field of exercise oncology have resulted in a growing body of evidence demonstrating physical activity and exercise interventions conducted among cancer survivors have a positive effect. As a result, there are increasing calls from cancer patients, survivors and health professionals for exercise programmes to be translated into community health and fitness settings [4]. Traditionally, participation in exercise oncology research includes rigorous assessment of study eligibility, medical screening, and no participant autonomy regarding the frequency, intensity, type and total duration of physical activity undertaken during the intervention period. Exercise programmes conducted within the community naturally overcome these issues and provide a real-world alternative to clinically delivered exercise interventions. However, such programmes must undergo rigorous evaluation in order to establish the effect of the programme and provide insight towards factors which limit accessibility and benefit. Established principles of knowledge-to-action translation and evaluation frameworks should be applied to better determine features of exercise programmes which make them successful and sustainable [41]. As first crucial step to ensuring community based exercise programmes for young adults with cancer are accessible efforts to make health professionals aware of existing lifestyle resources and exercise programmes should be made [42].

It is important that future work looks to evaluate the dose-response effect of exercise programmes conducted among young adult cancer survivors and establish the optimal number of exercise sessions per week or intensity of exercise required to have benefit. In addition, both cancer patients (those receiving treatment) and survivors (those who had completed treatment) were included within the intervention and grouped within the analyses. Future work should look to evaluate whether the effects of exercise during active cancer treatment differ to the effects observed post-treatment. This would provide some insight on whether early rehabilitation interventions initiated during the active treatment phase lead to better patient outcomes than interventions delivered in the survivorship phase once treatment has ended. To prevent loss-to-follow-up, greater insight towards geographical, psychosocial and socioeconomic determinants of programme uptake and attrition is required alongside studies evaluating the maintenance of PA behaviour change over time.

## Conclusion

The findings of the current study provide evidence of the physical and psychological benefits of the RENEW exercise programme delivered by Trekstock. The high uptake and engagement with the programme, combined with the data on patient reported outcomes, support the implementation of the RENEW exercise referral scheme across other cities within the UK. Given the growing body of evidence demonstrating exercise programmes can improve the length and quality of cancer survival [43], reduce co-morbidities associated with cancer treatment and foster resilience among cancer survivors, programmes such as RENEW have the potential to address some of the most pressing issues facing young people living with cancer.
